# A note on Striemer and Danckert's theory of prism adaptation in unilateral neglect

**DOI:** 10.3389/fnhum.2013.00044

**Published:** 2013-02-21

**Authors:** Styrmir Saevarsson, Árni Kristjánsson

**Affiliations:** ^1^Clinical Neuropsychology Research Group (EKN), Bogenhausen University HospitalMunich, Germany; ^2^Department of Psychology, University of IcelandReykjavik, Iceland; ^3^Institute of Cognitive Neuroscience, University College LondonLondon, UK

The nature of the therapeutic effects of prism adaptation (PA; see Striemer and Danckert, 2010a for a description) is a major point of controversy in clinical and neurocognitive psychology (e.g., Saevarsson et al., 2009, 2010). A detailed understanding of these effects could greatly advance the treatment and assessment of unilateral neglect. Many authors have concluded that perceptual aspects of neglect, such as visual perception and higher-order visuospatial cognition, improve following PA (e.g., Pisella et al., 2006; Redding and Wallace, 2006; Serino et al., 2006; Saevarsson et al., 2009, 2011; Nijboer et al., 2010, 2011). In contrast to this mainstream view, Striemer and Danckert (2010a) recently proposed that PA produces beneficial effects on spatial and premotor neglect (PMN; defined as the bias of movement from the ipsilesional to the contralesional side, most commonly reported for hand movements; Watson et al., 1978; Bisiach et al., 1990). For example, patients may have difficulties with initiating contralesional directional movements (directional hypokinesia; see Saevarsson, in press for an overview). Striemer and Danckert (2010b) argued that PA has little or no effect on perceptual biases and that only a few PA studies do, in fact, address perceptual neglect directly. Those that do, they claim, show only limited improvement following PA, especially in tasks requiring more explicit perceptual judgments than standard clinical neglect tests. Striemer and Danckert (2010a) also support their view by referring to plasticity changes in areas in the dorsal visual stream that are sometimes spared in neglect, such as the superior parietal lobule and the intraparietal sulcus. These areas are responsible for visuomotor behavior and attention, but are not involved in “more explicit perceptual judgments” (Striemer and Danckert, 2010a, p. 308), and are believed to play a major role in PA. In line with this, they also refer to studies where PA has been found to improve pointing and eye movements (Dijkerman et al., 2003; Ferber et al., 2003; Angeli et al., 2004; Serino et al., 2006).

We argue, however, that the evidence (Striemer and Danckert, 2010a,b) base their conclusions on can be interpreted differently. Visual neglect is in many cases accompanied by PMN although the motor response deficits of neglect may appear on their own (Goodale et al., 1990; Làdavas et al., 1993; Saevarsson, in press). It has proven to be more difficult to differentiate between the two than is often claimed because of various methodological problems (e.g., Mattingley and Driver, 1997). For example, a recent PA study by Striemer and Danckert (2010b) is based on the logic that motor and sensory components can be differentiated with standard neglect tests (see also Fortis et al., 2011). They measured motor components in three neglect patients with the landmark and line bisection tests. Their main finding was a reduced ipsilesional bias on-line bisection but not on the landmark task. This conclusion was based on the assumption that by requiring manual as well as verbal responses, visual, and premotor components of neglect could be isolated. We argue, however, that this is not as straightforward as claimed since these tests involve both contralesional visual input and eye movements even when responses are made verbally. Difficulties of many patients with shifting their gaze to the contralesional side (straight-ahead viewing bias; Ebersbach et al., 1996; Kim et al., 1997; see Beis et al., 1999 for evidence for improved gaze in neglect following hemifield eye-patching) is an important factor in this context. The two types of neglect, in other words, are conflated in the tasks. We note that performance on standard neglect tests can be interpreted in various ways and has been found to be inconsistent within the same PMN patient group (e.g., Harvey et al., 2002). For instance, an item on the contralesional side may be neglected because of difficulties with reaching to the contralesional side, eye movements to the affected side (less contralesional stimuli are foveated), or simply due to a lack of visual awareness of the contralesional side (e.g., Mattingley and Driver, 1997). The bisection and landmark tests do not distinguish between these sources of performance deficits. We argue, in other words, that standard neglect tests are as much tied to motor behavior as they are to visual processes. Uncoupling the two with standard tests may be impossible because of assessment issues such as whether visual neglect is accurately controlled for or not, related sensory and motor deficits and the role of cognitive load (see Saevarsson, in press). Importantly, Mattingley and Driver (1997) concluded that improved PMN may directly lessen symptoms of perceptual neglect because of more efficient feedback from eye movements, and that intact visual input may reduce PMN. In the four PA studies on motor function in neglect that Striemer and Danckert cite in support of their argument (Dijkerman et al., 2003; Ferber et al., 2003; Angeli et al., 2004; Serino et al., 2006), movements and visual input were not independently controlled for, and their independent roles (passive or active) in improved motor behavior are therefore unclear. For example, Dijkerman et al. (2003) argues that their patients suffered from visual neglect based on their performance on standard tests that are also sensitive to PMN components as discussed earlier.

Striemer and Danckert (2010a, p. 311) argue that there is little evidence for any effect of PA on “real-world” function, noting that “previous work has failed to observe significant effects of PA upon serial visual search tasks that measure attention in what could be considered a more ‘real-world’ scenario.” But this claim is directly contradicted by a recent study by Vangkilde and Habekost (2010) who tested visual search performance following PA in a complex scene (the “where's Waldo” task) in addition to a task where patients were placed in front of a cupboard containing a number of items and were asked to find particular ones. PA resulted in robust and consistent long-term improvements in the performance of both tasks (see Saevarsson et al., 2009, 2010; Saj et al., 2013).

Despite considerable progress, many unanswered questions remain regarding the neuroanatomy of motor and sensory components of neglect. For instance, Saevarsson (in press) analyzed 30 PMN studies and found that PMN is connected to various right-hemisphere and right-subcortical lesions that are commonly damaged in this affliction, such as frontal, parietal, and thalamus, among other structures. It is therefore not known whether PA improves PMN in patients where areas of the dorsal stream are spared, since these areas might not play an important role in the proposed interaction between PA and the motor response components of neglect. It is not clear whether it can be determined from lesion location whether patients suffer from PMN or not, and whether modulations of certain dorsal areas or the existence of certain lesions can explain corrected motor function in neglect following PA.

Although the effects of PA on neglect have been heavily studied over the last 15 years, the underlying mechanisms are still not fully understood. The evidence reviewed by Striemer and Danckert (2010a) seem to support their motor PA theory to some extent, although other interpretations are possible and further extensions are needed. Motor components of neglect and their relation to PA need to be investigated systematically, with controls for vision or other types of perception, along with careful study of the underlying neuroanatomy. In other words, PA experiments based on advanced assessment of motor and sensory components and statistical voxel-by-voxel lesion mapping are likely to provide more detailed information about the exact nature of any therapeutic effects of PA therapy on neglect. While in many ways we agree with Striemer and Danckert (2010a), our proposal is that PA corrects spatial premotor components (e.g., reaching from the ipsilesional to the contralesional side) in neglect, while visual neglect plays a passive role in preventing or reducing de-adaptation effects when neglect patients are confronted with their environment. This means that improved motor actions such as eye and hand movements are likely to last longer if the patient suffers from visual neglect as well as PMN. In other words, the better the visual awareness, the faster the de-adaptation will be and the fewer errors will occur, and vice versa (e.g., Michel et al., 2003, 2007; Goedert et al., 2010; Aimola et al., 2012). The lack of significant visual neglect or PMN might therefore explain the lack of consistent clinical effects. A number of other findings support this proposal. For example, Cubelli et al. (1991) found reduced directional hand deficits of visual neglect patients when blindfolded; the performance of many patients when pointing straight ahead when blindfolded improved compared to when they made similar pointing movements without a blindfold (see Làdavas et al., 1993 for discussion). This finding underlines the need to control for visual components when PA is used as an assessment tool. Evidence indicating considerable high comorbidity of visual neglect with PMN and a likely lack of isolated PMN cases may support the role of visual neglect in PA (Saevarsson, in press). Lee and Donkelaar (2006) found slowed PA in healthy subjects when subjects' pointing movements were completely visible and their premotor cortex was stimulated with TMS; but when only the endpoint of the movement was visible, PA occurred faster. This highlights the important role of passive on-line movement corrections of intact visual awareness in healthy observers and the potential importance of parietal lesions in PMN and premotor areas for PA. Furthermore, Saevarsson et al. (2008) found that right hemifield patching that is applied simultaneously with PA strengthens the effects on neglect compared to combined left hemifield patching and PA. This falls in the line with the proposed role of visual neglect in de-adaptation during PA since a combination of right patching and PA prevents visual feedback from the presumably non-affected visual field and forces adaptation to the affected hemifield. The adaptation may therefore be stronger and faster compared to when it is based on feedback from the “intact” visual field. It is also important to note that most studies report only the general effects on unilateral neglect, which obscures the symptom heterogeneity of subgroups such as PMN patients. For instance, the open-loop paradigm that is based on straight forward pointing while blindfolded is not particularly sensitive to PMN symptoms because it does not require contralesional reaching *per se*. Lack of exact diagnoses, experimental task differences, and neuroanatomical differences (e.g., Saevarsson et al., 2009 for lesion mapping evidences) between experimental groups may explain a considerable number of non-significant or controversial findings (e.g., Morris et al., 2004; Saevarsson, 2009; Saevarsson et al., 2009). Furthermore, using PA on healthy subjects has proved to be problematic since the effects have been found to be small or non-significant on different visuomotor tests, although short-lived adaptation has been found with pointing movements in the open-loop task (e.g., Morris et al., 2004; Michel, 2006; see Saevarsson, 2009 for a series of studies on healthy subjects). Interestingly, these findings have been attributed to intact visual awareness or lack of unilateral neglect.

The conclusion that visual or perceptual aspects of neglect are not part of successful PA treatment, in our opinion, is premature. What Striemer and Danckert's (2010a, p. 311) analysis correctly highlights is how heterogenous symptoms are between individual patients: “Just as the neglect syndrome is heterogenous and highly variable in presentation, the influence of PA on neglect could also be heterogenous and variable across patients.” This gets at the heart of the matter and could, in fact, explain why larger controlled trials fail to reveal clear effects at the group level. The heterogeneity in lesions and symptoms and various assessment complications prevent generalization. Striemer and Danckert (2010a) are right in pointing out how motor and visual neuroanatomical aspects of neglect are confounded in PA. It is for this reason that the conclusion that PA improves motor function without major influences of vision is inaccurate, given the current evidence. In conclusion, we feel that the evidence Striemer and Danckert's proposal (2010a) is not compelling enough. Advanced PMN diagnosis and lesion mapping with respect to PA is needed before definitive conclusions can be drawn regarding their hypothesis.
